# Supplementary material for: Empirical methods for controlling false positives and estimating confidence in ChIP-Seq peaks
Source: BMC Bioinformatics. 2008 Dec 5;9:523. doi: 10.1186/1471-2105-9-523 (PMC2628906; doi:10.1186/1471-2105-9-523)
Supplement: Additional File 1 — A variety of html documents from the USeq web site detailing the available applications, their best usage, output file type descriptions, command line menus, etc. [file 1471-2105-9-523-S1.zip › USeqUserGuides/JavaDoc.zip!/JavaDoc/bioroot/CronArchive.html]

CronArchive


|  |  |  |
| --- | --- | --- |
| **Overview** \| **Package** \| **Class** \| **Use** \| **Tree** \| **Deprecated** \| **Index** \| **Help** | |  |
| **PREV CLASS**   **NEXT CLASS** | **FRAMES**    **NO FRAMES**     **All Classes** |  |
| SUMMARY: NESTED \| FIELD \| CONSTR \| METHOD | DETAIL: FIELD \| CONSTR \| METHOD |  |


---


## bioroot Class CronArchive

```
java.lang.Object
  bioroot.CronArchive
```

---

``` public class CronArchive extends java.lang.Object ```

Emails lab group's collections to superusers.

---

**Constructor Summary**

`CronArchive()`


| **Method Summary** | |
| --- | --- |
| `static LabGroup[]` | `getLabGroupsToArchive(DBUtil bioRoot)`             Identifies lab groups that should be archived based on their last archive date and schedule. |
| `static java.lang.String` | `getMessage(LabGroup labGroup, boolean noRecords)` |
| `static void` | `loadSuperUserEmailAddresses(DBUtil bioRoot, LabGroup[] labGroups)`             Loads an array of LabGroup[] with superuser email addresses. |
| `static void` | `main(java.lang.String[] args)` |
| `static java.io.File` | `makeArchive(DBUtil bioRoot, UserBean superUser)`             Returns null or a zip file containing a labGroup's full archive. |
| `static boolean` | `setLastArchiveDate(LabGroup[] labGroups, DBUtil bioRoot)`             If archived will set that labgroups lastArchive to today |

**Methods inherited from class java.lang.Object**

`equals, getClass, hashCode, notify, notifyAll, toString, wait, wait, wait`

**Constructor Detail**

### CronArchive

```
public CronArchive()
```


**Method Detail**

### setLastArchiveDate

```
public static boolean setLastArchiveDate(LabGroup[] labGroups,
                                         DBUtil bioRoot)
```

:   If archived will set that labgroups lastArchive to today

---


### makeArchive

```
public static java.io.File makeArchive(DBUtil bioRoot,
                                       UserBean superUser)
```

:   Returns null or a zip file containing a labGroup's full archive.

---


### getMessage

```
public static java.lang.String getMessage(LabGroup labGroup,
                                          boolean noRecords)
```

---


### loadSuperUserEmailAddresses

```
public static void loadSuperUserEmailAddresses(DBUtil bioRoot,
                                               LabGroup[] labGroups)
```

:   Loads an array of LabGroup[] with superuser email addresses.
    Must be ordered by labGroup.id.

---


### getLabGroupsToArchive

```
public static LabGroup[] getLabGroupsToArchive(DBUtil bioRoot)
```

:   Identifies lab groups that should be archived based on their last archive date and schedule.
    Ordered by labGroup.id.

---


### main

```
public static void main(java.lang.String[] args)
```


---


|  |  |  |
| --- | --- | --- |
| **Overview** \| **Package** \| **Class** \| **Use** \| **Tree** \| **Deprecated** \| **Index** \| **Help** | |  |
| **PREV CLASS**   **NEXT CLASS** | **FRAMES**    **NO FRAMES**     **All Classes** |  |
| SUMMARY: NESTED \| FIELD \| CONSTR \| METHOD | DETAIL: FIELD \| CONSTR \| METHOD |  |


---
